# A Novel Bio-carrier Fabricated Using 3D Printing Technique for Wastewater Treatment

**DOI:** 10.1038/srep12400

**Published:** 2015-07-23

**Authors:** Yang Dong, Shu-Qian Fan, Yu Shen, Ji-Xiang Yang, Peng Yan, You-Peng Chen, Jing Li, Jin-Song Guo, Xuan-Ming Duan, Fang Fang, Shao-Yang Liu

**Affiliations:** 1 Key Laboratory of Reservoir Aquatic Environment of CAS, Chongqing Institute of Green and Intelligent Technology, Chinese Academy of Sciences, Chongqing 400714, China; 2 Key Laboratory of the Three Gorges Reservoir Region’s Eco-Environments of MOE, Chongqing University, Chongqing 400045, China; 3 Department of Chemistry and Physics, Troy University, Troy, AL 36082, USA

## Abstract

The structure of bio-carriers is one of the key operational characteristics of a biofilm reactor. The goal of this study is to develop a series of novel fullerene-type bio-carriers using the three-dimensional printing (3DP) technique. 3DP can fabricate bio-carriers with more specialized structures compared with traditional fabrication processes. In this research, three types of fullerene-type bio-carriers were fabricated using the 3DP technique and then compared with bio-carrier K3 (from AnoxKaldnes) in the areas of physicochemical properties and biofilm growth. Images acquired by 3D profiling and SEM indicated that the surface roughness of the 3DP bio-carrier was greater than that of K3. Furthermore, contact angle data indicated that the 3DP bio-carriers were more hydrophilic than K3. The biofilm on the 3DP bio-carriers exhibited higher microbial activity and stronger adhesion ability. These findings were attributed to excellent mass transfer of the substrate (and oxygen) between the vapour-liquid-solid tri-phase system and to the surface characteristics. It is concluded that the novel 3DP fullerene-type bio-carriers are ideal carriers for biofilm adherence and growth.

Three-dimensional printing (3DP) is a new technology used in the rapid prototyping (RP) industry. It is fundamentally a layer-by-layer fabrication process, in which the 2D cross-sectional profile of an object is determined by a computer model and printed in a layer of powder via deposition of a suitable binder. Successive 2D profiles are subsequently printed on freshly laid powder layer until the whole model object is completed^1^,^2^. Sanchs and Haggerty invented the 3DP technique at the Massachusetts Institute of Technology in 1991^3^. Over the subsequent two decades of development, this technique has been improved through the incorporation of many novel materials and method, and it is now widely used in numerous fields such as aerospace engineering, biomedical prototyping, pharmaceutical engineering, and process design^4^,^5^,^6^.

The 3DP technique offers many advantages over other manufacturing techniques. Traditional manufacturing methods depend on cutting and moulding technologies to create a limited number of structures and shapes, with more intricate hollow objects requiring the assembly of multiple separate parts. However, the 3DP technique transforms this process—3D printers can create many complex figures based on virtual designs constructed by computer-aided design (CAD), and the results are constrained only by a person’s imagination. This method also provides better structural integrity and durability. The 3DP technique can remove the limitations in combining different raw materials, a problem that can arise in traditional methods when discrepancies exist between chemical and physical properties. The process of the 3DP technique enables rapid automated manufacturing^7^. In the long term, the range of industrial 3DP technique applications will skyrocket, as the newest 3DP techniques accommodate larger products objects and achieve greater levels of precision at lower cost^8^. The 3DP technique is one of the most viable rapid prototyping technologies and has an enormous potential application value.

Biofilm reactors are one of the most important technologies being widely used in wastewater treatment plants. Similar to activated sludge systems, they can provide organic matter removal, nitrification, denitrification and phosphorus removal^9^. Bio-carriers, one of the key components used in wastewater treatment, can enrich microorganisms at the surface to improve the amount of biomass in the reactor. Currently, there are many types of available bio-carriers having different shapes and sizes; they are made of polyethylene, polypropylene or high-density polyethylene with a typical density slightly less than water^10^. To obtain better organic matter removal and to improve mass transfer efficiency in reactors, multiple research projects involving bio-carriers have been conducted^11^,^12^,^13^. For example, Ahmed Eldyasti compared the influence of particle properties on biofilm structure, reactor performance, and energy consumption for denitrifying fluidized bed bioreactors using maxi-blast plastic, multi-blast plastic, natural zeolite, and lava rock^14^. D.J. Gapes compared two types of bio-carriers (Kaldnes K1 and Natrix C10/10) with different structures in terms of their internal and external mass transfer resistance when a biofilm was present. The bio-carrier structure was determined to be the controlling factor for process performance in laboratory-scale nitrifying of suspended carrier reactors^15^.

The 3DP technique provides new opportunities to change the characteristics of bio-carriers in novel ways because of the separation in 3D model design and processing. After the model is established on the computer, the shape and size of the bio-carriers can be designed and controlled to increase effective specific surface area and promote mass transfer. Material selection determines the density and robustness of the bio-carriers, as well as the affinity between carrier and microorganism. In this study, a series of fullerene-type bio-carriers was fabricated using the 3DP technique and compared with one of the most common bio-carriers to determine their chemical and physical properties and evaluate biofilm growth performance.

## Materials and Methods

### Design and fabrication of 3DP bio-carriers

The design inspiration for these bio-carrier structures is the molecular structure of fullerene. The basic structure of the fullerene-type bio-carrier is modelled by mapping the topological structure of fullerene to a sphere. The basic structure is then hollowed to provide locations for biofilm growth. For performance reasons, a series of fullerene-type bio-carriers was designed. For example, to enhance bio-carrier strength and maximize specific surface area, various numbers of ribs were incorporated into the honeycomb-like cellular interior.

The novel bio-carriers were fabricated from nylon with laser selective sintering (SLS) based on the 3DP technique. The Nylon material FS3200PA was purchased from Hunan Farsoon High-tech Co., Ltd, China, with bulk density 0.45 g/cc and melting point 183 °C. The SLS equipment FARSOON 251, also provided by Hunan Farsoon, was used to fabricate the bio-carriers with the standard 3DP process.

### Bio-carrier chemical properties

For measurement of the zeta potential, 0.01 g of the material was ground into powder and dispersed in 10 mL of deionized water to form a colloidal solution. The samples were allowed to stand for 5 min to let larger particles settle. An aliquot was processed by a ZatasizerNanoZS particle size analyser (Malvern Instruments Ltd Co), which automatically calculates the electrophoretic mobility of the particles and converts it to the zeta potential using the Smoluchowski equation. Contact angles were obtained using the sessile drop method with a contact angle analyser (DSA1000, KRUSS GmbH). A NewView^TM^ 7100 white light interferometer (ZYGO Corporation Co.) was used to acquire a 3D profile image of the surface of the K3 and 3DP bio-carriers. The white light interferometer uses a special optical configuration and short-coherence-length light sources that optimize the interaction between the reflected light from the sample and the reference beam. This process characterizes and quantifies surface roughness and other topographical features with excellent precision and accuracy. Material compositions of the materials was analyzed using an ATR spectrophotometer (Agilent Cary 630, Agilent Technologies Co).

### Biofilm reactor configuration

**Figure 1:**
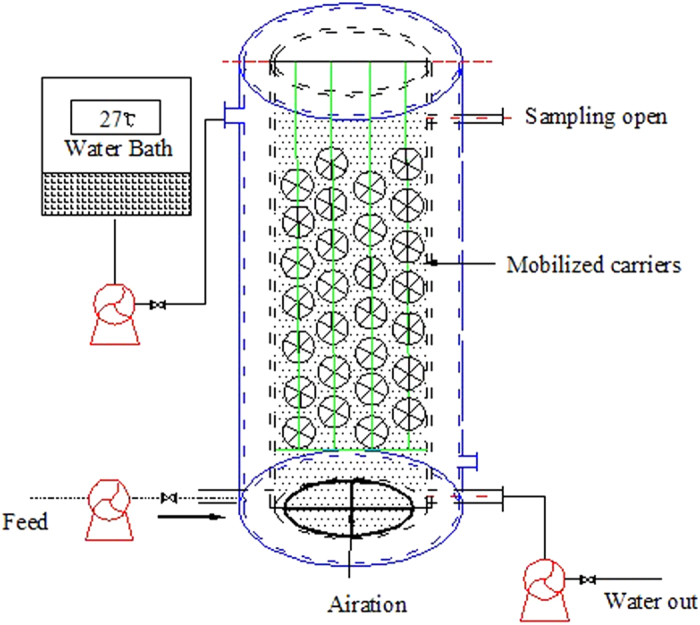
Schematic of the sequencing biofilm batch reactor containing different types of bio-carriers.

A laboratory-scale batch reactor was constructed from commercially available Plexiglas vessels with a working volume of 22.6 L and internal dimensions of 24 cm (diameter) × 50 cm (height). As shown in Fig. 1, the reactor contained two regions: an outside water bath thermal insulation layer to maintain the temperature at 27 °C, and an inner reaction zone. The effective reactor volume was 8.74 L. The bio-carriers were fixed onto the lines one by one with identical spacing and were suspended in the reaction zone. The packing rate was 40% and the void ratio was 92%. A nanometre aeration tube was installed at the bottom of the reactor to ensure sufficient gas dispersion in the liquid phase.

### Reactor start-up and operation

The reactor was inoculated with activated sludge acquired from Beibei Municipal Wastewater Plant, Chongqing, China, which had a mixed liquor suspended solids (MLSS) content of 11.275 g/L, and an SV (Settling Velocity) of 82. The mixed liquor from the secondary sedimentation tank was inoculated at a ratio of 1:3 (v/v) with reactor volume, and the reactor was operated with designed synthetic feed to support biomass formation on the bio-carriers. After aeration for 48 hours with no influent or effluent, all activated sludge was drained out of the reactor. The simulated sewage was then pumped into the system in batches. A start-up period of sequencing discontinuous operation was conducted to form the biofilms. In the first stage (5 days), the biofilm- configured system was operated under aerobic metabolic mode with a total cycle period of 8 h (retention time) consisting of 15 min of fill phase, 6.5 h of reaction (aerobic) phase with recycling, 15 min of out phase and 1 h of decant time. The controller was programmed to operate on a repeating 8-hour cycle. In the second stage (from day 5 to day 12), the biofilm-configured system was operated under aerobic metabolic mode with a total cycle period of 6 h (retention time) consisting of 15 min of fill phase, 4.5 h of reaction (aerobic) phase with recycling, 15 min of out phase and 1 h of decant time. In the third stage (from day 12 to day 45), the biofilm-configured system was operated with a total cycle period of 4 h (retention time) consisting of 15 min of fill phase, 2.5 h of reaction (aerobic) phase with recycling, 15 min of out phase and 1 h of decant time.

### Simulation of sewage

An activation solution was used to simulate real sewage. It consisted of 500–600 mg COD (Chemical Oxygen Demand) g/L, 25–30 mg NH_4_-N/L, 5–6 mg TP/L, and 0.1% (v/v) of a trace nutrient solution containing the following (g/L): FeCl_2_·4H_2_O 1.5; CoCl_2_·6H_2_O 0.190; ZnCl_2_ 0.07, MnSO_4_·H_2_O 0.080, NiCl_2_·2H_2_O 0.024, and H_3_BO_3_ 0.006.

### Characterization of the biofilm

#### Biomass and thickness measurement

To evaluate the biomass supported by bio-carriers, samples of bio-carrier elements were taken from the reactor. Biofilm solids were determined by the difference in weight of dried bio-carriers (105 °C for ≥1 h) before and after biofilm removal. Removal of biofilm solids was performed in NaOH (4 M) (Analytical grade, purchased from Chemical Reagent Company in China,) through mechanical shaking and ultrasonication under 60 °C. The SS (Suspended Substance) of the biofilm was measured directly according to Chinese SEPA Standard Methods^17^. A subset of the bio-carriers was selected for biofilm thickness measurements. Bio-carriers with attached biofilm were carefully cut into several sections, and biofilm thickness was measured with a pair of vernier calipers^18^. Because of the thickness heterogeneity of the biofilm on the bio-carriers, 10 measurements were averaged to determine the biofilm thickness.

#### Measurement of specific oxygen uptake rate (SOUR)

SOUR is an important parameter to express the microbial activity of biofilm in the wastewater treatment process. One bio-carrier was removed from the reactor and inserted into a flask containing 500 mL of the simulated wastewater, which was aerated to saturate the dissolved oxygen (DO). Aeration was stopped and the DO variation with time was monitored using a DO metre (METTLER TOLEDO SG9, Mettler-Toledo International Inc. Co). The value of SOUR per gram biomass was calculated according to the following equation:


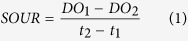


Where SOUR is oxygen consumed over unit time (mg O_2_L^−1^S^−1^); DO_1_ is DO concentration at time t_1_ (mgL^−1^); DO_2_ is the DO concentration at time t_2_ (mgL^−1^); and t(s) is the measuring time^19^.

### Analytical method for wastewater

The performance of the reactor was assessed by monitoring COD and ammonium nitrogen (NH_4_^+^-N) removal efficiency throughout the operation. Both of these parameters were analysed according to standard methods for examination of water and wastewater^17^. All experiments were conducted in duplicate and average values were used for data analysis.

## Results and Discussion

### Bio-carrier parameters

**Figure 2:**
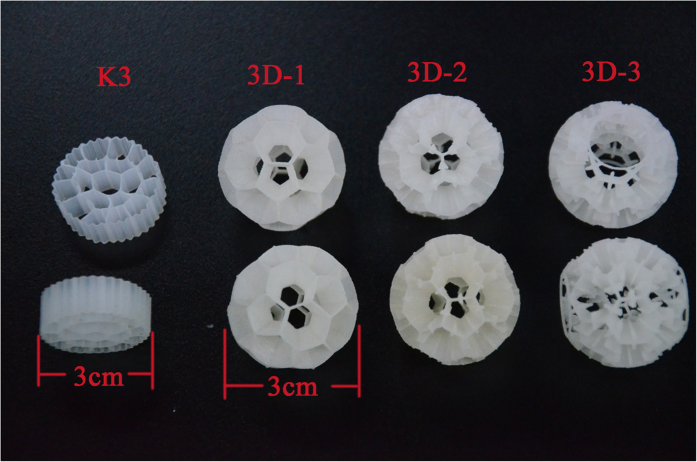
The 4 types of bio-carriers used in the bioreactor (photograph by Yang Dong) .

Figure 2 shows the shape of the four types of bio-carriers. The porous biofilm carrier (AnoxKaldnes, K3 bio-carriers) used in this study had a cylindrical shape (dimensions of 30 mm in radius and 8 mm in height). It was made of polyethylene with a relatively large surface area of 800 m^2^/m^3^ per element and a density of 0.98 g/cm^3^ 16.

Three fullerene-type bio-carriers were fabricated in this study. They had a cellular globular structure built up of mostly polyhedral, which constituted the basic pattern used for spherical mapping in the geometrical transformation. They consisted of a three-dimensional hollow honeycomb spherical biological filler structure formed from pentahedrons and hexahedrons. The diameter of the 3DP bio-carriers was 3 cm. 3D-2 bio-carriers contained rib mediators with a biological-type structure. 3D-3 bio-carriers also contained rib mediators but had empty inner sides. The design of the 3D-3 bio-carriers, which featured a larger surface area and empty interior, was intended to promote substrate and oxygen transfer into the biofilm. Moreover, this bio-carrier configuration was expected to shed dead microorganisms more easily.

The porosity of the three fullerene-type bio-carriers was greater than 90%, and their surface roughness average (RA) was greater than 70 um. Their mechanical properties matched those of the K3 bio-carrier; specifically, their tensile strength exceeded 30 MPa, their bending strength exceeded 20 MPa, and their impact strength exceeded 3 KJ/m^2^.

### Bio-carriers properties

#### Basic physical parameters

As specified in Table 1, the density of all four carriers was close to the water to ensure that they would circulate easily in water. The specific surface is the critical parameter required guarantee satisfactory mass transport in the bio-carriers. Of all carriers, the specific area of the K3 bio-carriers was the largest (as high as 800 m^2^/m^3^), followed by the 3D-3, 3D-2 and 3D-1 carriers. The larger rib mediators on the pentahedron and hexahedron surfaces provided the 3D-3 carriers with more surface area.

#### Surface morphology

**Figure 3:**
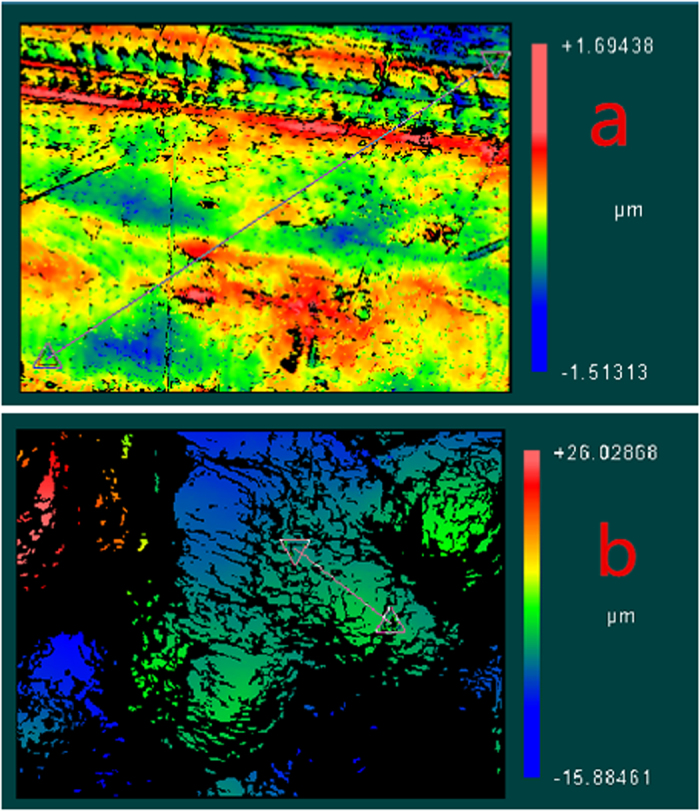
Surface 3D profile imaging of 3DP bio-carriers and K3 bio-carriers using profilometry: K3 (**a**) and 3DP bio-carriers (**b**).

**Figure 4:**
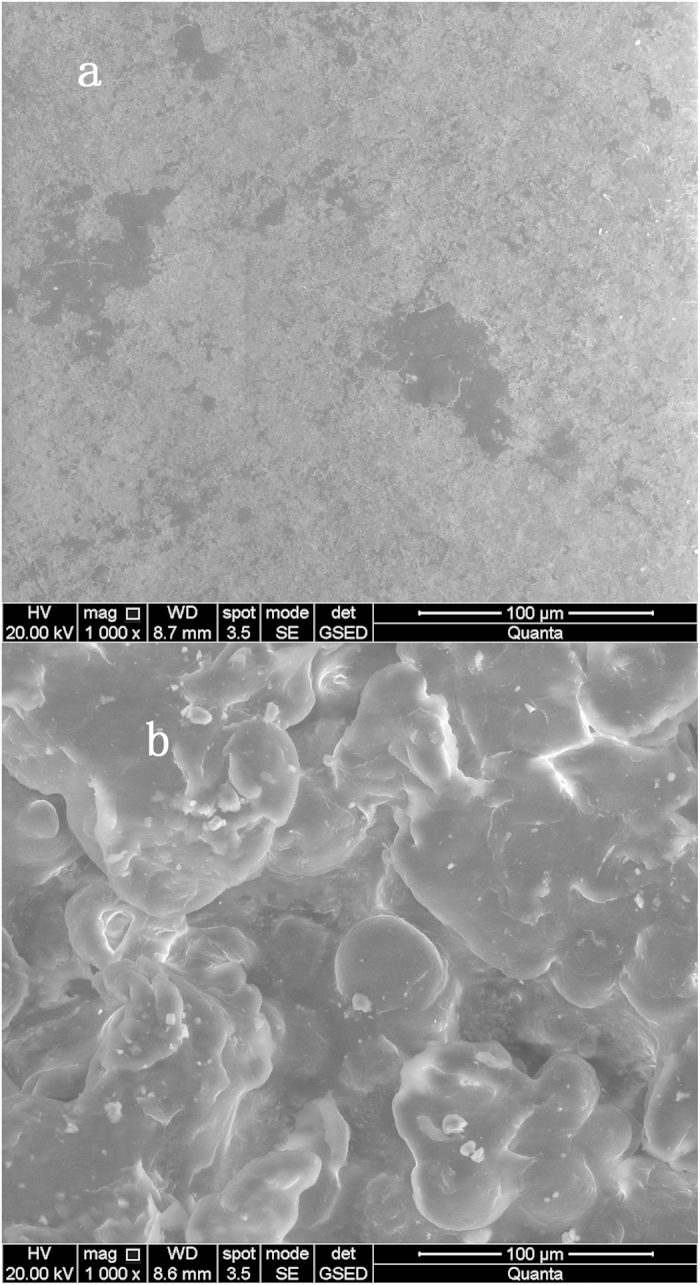
SEM photographs of the surfaces of K3 (**a**) and 3DP bio-carriers (**b**).

Representative surface topographical images of K3 and 3DP bio-carriers, obtained using white light interferometry, are shown in Fig. 3. The relative smoothness of the K3 material surface increase the reflected signal, as shown in Fig. 3a. The RA and root mean square (RMS) data was obtained as 0.247 μm and 0.319 μm, respectively. The peak valley (PV) range of the optical profilometer used here is 0.1 nm ~ 150 μm. Figure 3b shows only part of the 3DP bio-carrier surface, because in vertical scanning mode, the light was not reflected back sufficiently from the bottom of the material. The RA and RMS could not be fully obtained, as some values lay outside the range of 0.1 nm–150 μm. As measured by the surface profiler, the RA value of the 3DP bio-carrier surface was 70 μm. The morphology of each type of carrier material was investigated using ESEM at 4 kV. The relative degree of embossment on the surface was large, as shown by Fig. 4. The K3 carrier surface was relatively smooth (Fig. 4a). The ESEM images were in some certain agreement with the partial measurements recorded by the white light interferometer. It can be concluded that the surface roughness of the 3DP bio-carriers is higher than that of bio-carrier K3.

#### Hydrophilicity

**Figure 5:**
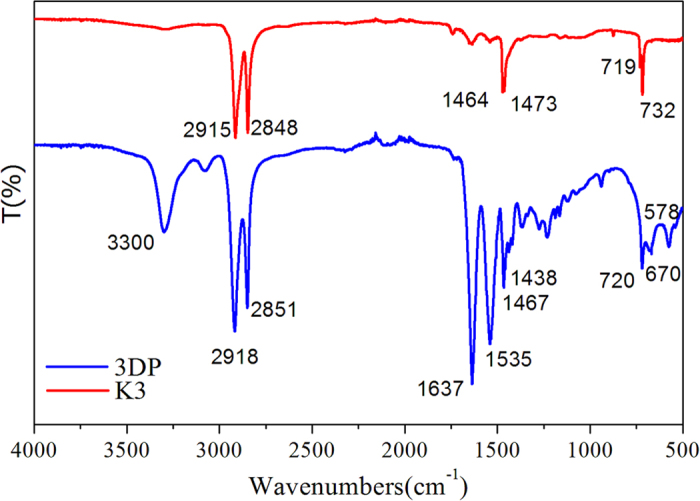
IR spectra of the K3 and 3DP bio-carriers.

The IR spectra of the K3 and 3DP bio-carriers are shown in Fig. 5. The IR bands were assigned according to the referenced literatures^20^,^21^. The large band at approximately 3300 cm^−1^, related to the O-H stretching mode of the hydroxyl functional groups, is shown in the graph for the 3DP material only. The bands at 2918, 2915, 2851 and 2848 cm^−1^ are assigned to C-H stretching vibrations, whereas those at 1464 and 1467 cm^−1^ were characteristic of C-H deformation vibrations. The intensity of O-H stretching vibrations in the 3D-printed material is greater than that in K3. The band at 1637 cm^−1^ is attributed to C=O stretching vibrations. The 3D-printed material had more functional groups compared with the K3 material. The bands in the 1188–1166 cm^−1^ region were correspond to C-O or C-C stretching vibrations in the 3D-printed material, whereas the band at 1438 cm^−1^ was assigned to C=O stretching vibrations. Unsaturated bonds were found in the region below 900 cm^−1^. The water contact angles of the K3 and 3DP materials were 50.7 ± 2.5 and 36.7 ± 3.1, respectively, which indicates that the 3DP material was more hydrophilic than the K3 material.

### Biofilm on the 3DP bio-carriers

#### Comparison of biofilm mass

**Figure 6:**
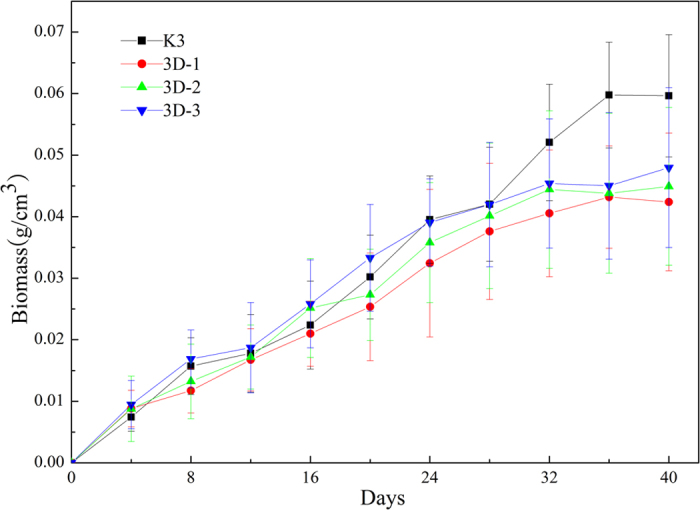
Biomass formed on the different carriers.

Figure 6 shows a comparison of the dry biomass values of the different bio-carriers as measured every four days, from the beginning (1st sampling, day 4) to the end (10th sampling, day 40) of the colonization experiment performed in the bioreactor system. By day 20, the average dry biomass of 3DP bio-carriers reached 0.0259 g/cm^3^, which is 9.5% higher than that of the K3 bio-carrier. Aside from the more hydrophilic nature of the 3DP material, this finding can be explained by the difference in surface roughness of group of the 3DP bio-carriers and the control (K3). After inoculation by the activated sludge in the reactor, the bacteria attached to the hydrophilic and rough surface more easily if they had a similar zeta position. The complex structure of the 3DP bio-carriers was able to retain more bacteria from the inoculated sludge. By day 36, the average dry biomass of the biofilm growing on K3 bio-carriers was as high as 0.0574 g/cm^3^, and is 24.8% higher than that of 3D-3 bio-carriers. Among the 3DP carriers, the mass of biofilm grew best on the 3D-3 bio-carriers and worst on the 3D-1 bio-carriers.

Two reasons can explain the difference of biomass between these bio-carriers. The first reason relates to their shape and size. All the bio-carriers were exposed to the same conditions of hydraulic loading and aeration. For the K3 bio-carriers, the biofilm could only grow on the inner surface, which was protected from the shear force arising from the aeration. Biofilm growth was limited on the outside surface of the bio-carriers because of the scouring force formed by aeration^16^. The 3DP bio-carriers, however, featured a three-dimensional hollow-type honeycomb consisting of a spherical biological filler structure with five or six sides, lacking protected areas. In the fixed status, the volume of 3DP bio-carriers was 3 times larger than that of K3, thus, the 3DP bio-carriers were exposed to more scouring force from the aeration. The other reason that the K3 bio-carrier processed more biomass is the specific surface area. The specific surface area of per K3 carriers is 33.3% larger than that of the 3DP bio-carriers, which indicated that K3 can could provide the largest support area for biofilm growth. These two reasons could explain why K3 supported the highest mass of biofilm^22^.

#### Comparison of biofilm thickness

**Figure 7:**
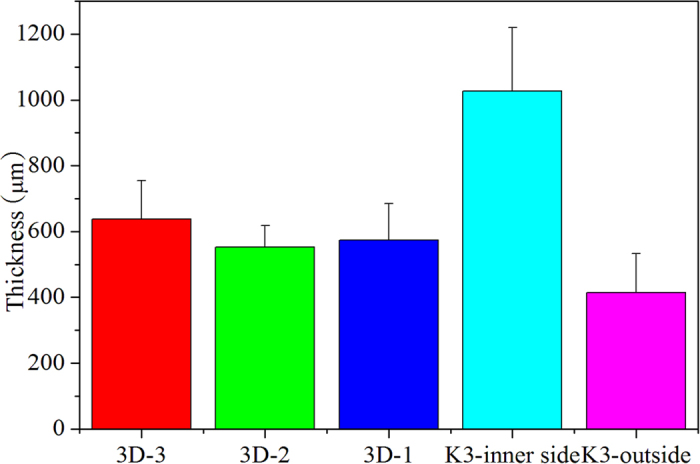
The thickness of biofilms attached to different bio-carriers on day 25.

The biofilm thickness was measured on day 25, when the biofilm was considered to be mature and stable. For the K3 bio-carriers, the biofilm mainly accumulated in the bio-carrier interspaces. The thickness of the biofilm growing on both surfaces of the K3 bio-carriers was measured separately. Figure 7 shows that the thickness of the biofilm attached to the different types of bio-carriers can be ranked as follows: K3 (inner side) >3D-3>3D-2>3D-1>K3 (outer side). The thickness of the biofilm attached to K3 was uneven, varying between 200 μm and 555 μm, and reaching 1026 μm at the interspace corners. For the K3 bio-carriers, the thickness of the biofilm grown in the inner side was much thicker than that grow on the outer side. Since the K3 bio-carriers were suffering from bubble wash, the most biofilm accumulated in the inner passage or compartments in the carriers. The inner passages are narrow in the K3 bio-carriers. Because the EPS (Extracellular Polymeric Substances) excreted by microorganism can aggregate microorganism and keep them together in a three-dimensional matrix, it was easier for the biofilms to adhere with each other in narrow and complicated passages or compartments^23^,^24^,^25^. For the spherical honeycomb bio-carriers, the thickness of the biofilm was relatively homogeneous, varying between 425 to 760, 485 to 630, and 610 to 825 μm for 3D-1, 3D-2, and 3D-3 separately. The ridges on 3D-3 were bigger than on 3D-2 (shown in Fig. 1), forming small and more complicated interspaces on the surface; therefore, it was easier for biofilm sheets to connect. This explains why the biofilms grew thicker on the 3D-3 bio-carriers compared to the other 3DP carriers.

Biofilm thickness is the principal parameter used to evaluate the substrate consumption rate in the biofilm. A previous study showed that a biofilm thicker than 700 μm cannot sustain nitrogen removal because of the lack of substrate in the deep anaerobic layer. The researchers found that 700 μm is the optimal thickness of a biofilm at a certain ammonium surface load^26^. Based on this finding, nitrogen removal by a biofilm would be more efficient on 3D-1 and 3D-2 bio-carriers.

#### Comparison of biofilm activity

SOUR was monitored at the end of the sequencing biofilm batch reactor (SBBR) aeration phase to assess the degradation ability of self-immobilized biofilms in an aerobic microenvironment. This parameter was measured on day 35, when the operation of the bioreactor was considered to be stable. SOUR values of the biofilms in the SBBR with different bio-carriers are shown in Table 2. The sequence of decreasing SOUR values is 3D-3, 3D-1, 3D-2 and K3. Differences in biofilm activity among the bio-carriers can be attributed to differences in bio-carrier structure and biofilm composition, which are affected by large changes in loading rate. The SOUR values of the biofilms were generally high during initial of bacterial growth under a high organic loading condition. Aerobic oxidation mainly occurred at the upper half of the biofilm during the SBBR aeration stage^26^. In the same growing environment, the biofilm that was attached to the inner surface of the K3 bio-carrier was thicker than on the 3DP bio-carriers, as shown in Fig. 7. A thick biofilm can impede the permeation of oxygen and substrate species into the biofilm and can reduce the dissolved oxygen concentration in microbial cells^27^. Furthermore, if the carrier elements were designed to have very large surface (>500 m^2^/m^3^ for the inner sides), the passages through the carrier element would be so narrow that it would be difficult to avoid inhibiting the mass transport^28^. The K3 bio-carriers has a relatively large surface area of 800 m^2^/m^3^ per element and the 3DP bio-carriers has a surface area of 437–600 m^2^/m^3^ per element. The hollow-type honey comb consisting of a spherical structure with five or six sides provides more inner space for mass transfer into the interior of bacteria. 3D-3 bio-carriers have a hollow-core construction, open to the outside. This design assures that the biofilm growing on the 3DP-3 bio-carriers maintains substance exchange with the external environment. As a result, the high microbial metabolism is maintained, and the bacteria can reach a higher activity expressed through the SOUR value for 3DP bio-carriers.

### Wastewater treatment performance of the reactor contained 3DP bio-carriers

**Figure 8:**
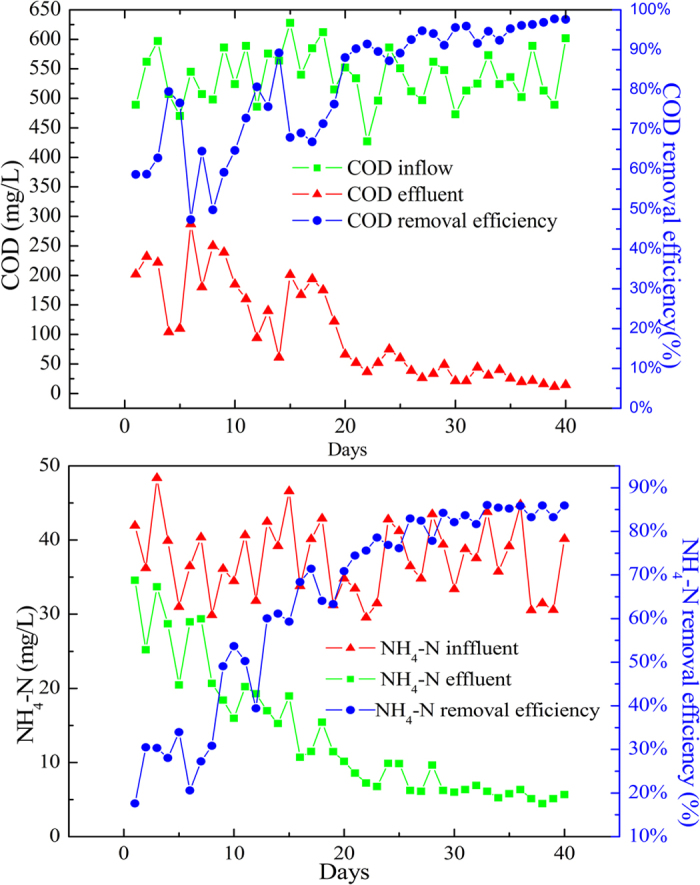
COD and NH_4_-N removal in SBBR for four types of bio-carriers.

The performance of the reactor was assessed by monitoring COD and NH_3_ removal efficiency throughout the operation. In Fig. 8, the SBBR was initially operated at an organic loading rate of 1.38 Kg COD/cum-day, and the performance of the reactor with respect to the COD and NH_4_-N removal efficiency was assessed during cycle operation. The reactor exhibited a maximum COD and NH_4_-N removal of 76.60% and 33.96% respectively in the first five days. The COD and NH_4_-N removal rates were relatively low during the initial phase of sequence batch operation; with increasing sequence time, more rapid COD and NH_4_-N removal was observed. After 4 h of cycle operation, maximum COD and NH_4_-N removal values of 91.6% and 83.68% were achieved and were thereafter nearly constant, indicating that the reactor was maintaining stable performance. The biofilm was considered to be mature during that period.

## Conclusions

In this research, three novel fullerene-type bio-carriers fabricated by a 3DP technique were developed to provide a growth environment for biofilm in wastewater treatment. This study shows that the hydrophobicity and roughness of the 3DP bio-carriers are higher than those of K3 bio-carriers. The biofilms growing on the 3DP bio-carriers were more homogeneous than those on the K3 bio-carriers. The thickness of the biofilms growing on the 3DP bio-carriers was thicker than that on the outside of K3, but thinner than the inside of K3. The SOUR values of biofilm grown on the 3DP bio-carriers were 8.73%–27.60% higher than K3 bio-carriers, indicating that the bioactivity of the biofilm attached to 3DP bio-carriers was higher. The 3DP bio-carriers offer certain advantages for biofilm growth by providing an appropriate microenvironment for bacterial growth in wastewater treatment. Whether 3DP bio-carriers can provide a good performance in the moving bed biofilm reactor as well deserves further investigation.

## Additional Information

**How to cite this article**: Dong, Y. *et al.* A Novel Bio-carrier Fabricated Using 3D Printing Technique for Wastewater Treatment. *Sci. Rep.* **5**, 12400; doi: 10.1038/srep12400 (2015).

## Figures and Tables

**Table 1:** Features of the 3DP and K3 bio-carriers.

	**K3**	**3D-1**	**3D-2**	**3D-3**
Volume (cm^3^)	3.925	14.13	14.13	14.13
Weight (g)	0.8756 ± 0.0595	1.2631 ± 0.1307	1.5953 ± 0.1875	2.1003 ± 0.1582
specific surface (Element volume1/m)	800	437	560	600
Density (g/cm^3^)	0.95	0.97	0.97	0.96
Zeta	−24.03	−29.45	−30.1	−28.76

**Table 2:** SOUR values of biofilms on bio-carriers.

	**K3**	**3D-1**	**3D-2**	**3D-3**
SOUR (mg O_2_L^−1^S^−1^)	30.36	34.45	33.01	38.74
